# Development and Characterization of a Chemically Defined Food for *Drosophila*

**DOI:** 10.1371/journal.pone.0067308

**Published:** 2013-07-02

**Authors:** Wen-Chih Lee, Craig A. Micchelli

**Affiliations:** Department of Developmental Biology, Washington University School of Medicine, St. Louis, Missouri, United States of America; VIB and KU Leuven, Belgium

## Abstract

Diet can affect a spectrum of biological processes ranging from behavior to cellular metabolism. Yet, the precise role of an individual dietary constituent can be a difficult variable to isolate experimentally. A chemically defined food (CDF) permits the systematic evaluation of individual macro- and micronutrients. In addition, CDF facilitates the direct comparison of data obtained independently from different laboratories. Here, we report the development and characterization of a CDF for *Drosophila*. We show that CDF can support the long-term culture of laboratory strains and demonstrate that this formulation has utility in isolating macronutrient from caloric density requirements in studies of development, longevity and reproduction.

## Introduction

Organisms must acquire nutrients from food to meet the energetic and metabolic requirements necessary for life. Deficiency or overabundance of dietary nutrients is a key physiological variable influencing developmental, homeostatic and disease processes [1]–[6]. Understanding how nutrient-dependent physiological status can influence cellular processes has been the subject of intensive investigation. For example, in *Drosophila*, dietary manipulation has been shown to broadly affect global transcriptional programs, as well as specific cellular processes such as the expansion of stem and progenitor cell lineages, maintenance of stem cell niches, development, regeneration, reproduction and longevity [7]–[18].

While these recent studies in *Drosophila* underscore the importance of diet-induced changes on cellular function, they have all employed standard complex (undefined) media as a means to manipulate dietary nutrients. Complex media is composed of ingredients of biological origin (e.g. yeast, cornmeal, molasses). Such ingredients are essentially nutrient composites that have different profiles depending on where and when they are sourced. Thus, an important limitation of the complex diet is that its composition is variable and difficult to precisely manipulate [19]. While diluting or excluding components of a complex media permits gross nutrient manipulation, it also introduces the confounding variable of altering caloric density (content).

A powerful tool to decipher the effects of diet is the use of chemically defined food (CDF) media, which consists entirely of purified compounds [20]–[22]. Notably, CDFs have only been fully developed in a limited number of experimental model organisms [23]–[25]. Such diets permit the systematic evaluation of individual macro- or micronutrients and facilitate the interpretation and replication of experimental data obtained independently by different investigators [26], [27]. In addition, use of CDF permits caloric density to be more tightly controlled.

Classic studies in *Drosophila* have determined the nutritional and metabolic requirements for the developing larvae. Essential components of the media include proteins, carbohydrates, lipids, nucleic acid, vitamins and salts [28]–[30]. Together, these studies provided a basis for establishing the first chemically defined media for larval culture [31]. In contrast, the dietary requirements for adults have been largely neglected since adults are capable of surviving on an energy source alone (e.g. sucrose) and because it has been assumed that nutritional requirements are similar during all stages of life. In this regard it is worth noting that certain nutritional requirements between larvae and adults can differ by two or three orders of magnitude [32], [33].

More recently, CDF recipes have been reported for adult *Drosophila* [17], [34]. However, previous formulations have been technically flawed [34], [35] or characterized only under a narrow set of conditions [17]. Consequently, the overall use and utility of CDF in *Drosophila* has remained rather limited. Here, we describe an open-source CDF suitable for long-term culture (>30 generations) of *Drosophila* laboratory strains. The effects of this CDF were analyzed at different stages of the *Drosophila* life cycle and compared to standard complex media. Finally, we used the CDF to directly test the requirement of individual dietary macronutrients on *Drosophila* development, reproduction and longevity.

## Materials and Methods

### Fly Strains

*w^1118^* flies were used for all feeding assays performed in this study. All experiments were performed at 25 degrees Celsius unless otherwise noted.

### Development of Chemically Defined Food (CDF)

CDF was formulated by optimizing the macro- and micronutrient components from several existing studies [17], [34]–[36]. The concentration of amino acids, ribonucleotides, metals and vitamins was based on the work of Troen et al. [34], [35]. We modified the amino acid composition of Troen et al. [34] to include amino acids that were previously excluded (see File S1). Both the composition and concentration of carbohydrates and lipids was based on the work of Grandison et al. [17]. The amino acid to carbohydrate energy ratio was set at 1∶4, a proportion shown to optimize overall fitness by Lee et al. [36]. CDF lipid levels were set at 2%. This value was chosen by surveying a series of standard recipes on the Bloomington Stock Center website (http://flystocks.bio.indiana.edu/Fly_Work/media-recipes/media-recipes.htm) with different lipid compositions and selecting the lipid level associated with best stock propagation (see File S1). Thus, the ratio of food energy per mass in CDF for amino acids, carbohydrates and lipids is 1∶4: 0.1, respectively. To derive the caloric density of CDF, we first estimated the caloric density from a series of standard complex food recipes referenced on the Bloomington stock center website. These fell in a range between 275–991 K-cal/L (see File S1). Troen et al. suggested that 300–400 K-cal/L was an optimal caloric density [34], [35]. We therefore focused on testing media with caloric density in the range of 100–500 K-cal/L (see File S1).

### Preparation of Chemically Defined Food

To simplify production of CDF we first created a series of three powered master mixes; essential amino acid mix (TD.10473); non-essential amino acid mix (TD.110036); and basal mix (TD.10475). See Table 1 and File S1 for additional details. These custom reagent mixes can be obtained from Harlan Laboratories, Inc., IN, US using the TD reference numbers indicated. Two additional stock solutions were prepared (see File S1): 1) 5X carbohydrate mix (autoclaved and stored at 4°C) and 2) A freshly prepared 100X slurry of lipid vortexed into water until no solids are visible. Commercial sources for all ingredients above are listed in File S1.

**Table 1:** Recipes for 400 K-cal/Liter chemically defined food (CDF^400K^) and regular food (RF).

Recipe for 400 K-cal/Liter chemically defined food (CDF^400K^).			
Ingredients	gram/Liter	Ingredients	gram/Liter
**Amino Acids**	**19.61**	**Vitamins, Minerals, and Nucleic Acids**	**3.20**
L-arginine HCl	1.67	Vitamin B12 (0.1% in mannitol)	0.01880
L-histidine HCl-H_2_O	0.47	Biotin	0.00002
L-isoleucine	0.81	p-Aminobenzoic Acid	0.00200
L-leucine	1.32	Inositol	0.04200
L-lysine HCl	2.78	Niacin	0.01000
L-methionine	0.58	Calcium Pantothenate	0.00599
L-phenylalanine	0.94	Folic Acid	0.00599
L-threonine	0.90	Pyridoxine HCl	0.00300
L-tryptophan	0.74	Riboflavin	0.00241
L-valine	1.28	Thiamin HCl	0.00151
L-alanine	1.11	Choline Bitartrate	0.03600
L-asparagine	0.53	Vitamin A Palmitate (500,000 IU/g)	0.00270
L-aspartic acid	0.53	Vitamin E, DL-alpha tocopheryl acetate	
L-cystine	0.43	(500 IU/g)	0.03300
L-glutamic acid	1.20	Vitamin D3, cholecalciferol (500,000 IU/g)	0.00067
L-glutamine	1.20	Vitamin K, MSB complex	0.00051
Glycine	0.43	Zinc Carbonate	0.01820
L-proline	0.90	Cupric Carbonate	0.00850
L-serine	0.98	Chromium Potassium Sulfate, dodecahydrate	0.00540
L-tyrosine	0.81	Potassium Phosphate, dibasic	0.60598
		Potassium Phosphate, monobasic	0.60598
**Carbohydrates**	**78.43**	Calcium Chloride	0.01291
Sucrose	63.68	Ferrous Sulfate, heptahydrate	0.01291
Glucose	5.93	Magnesium Sulfate, heptahydrate	0.24599
Lactose	4.92	Manganese Sulfate, monohydrate	0.00979
Trehalose	3.91	Sodium Chloride	0.01291
		RNA	0.99991
**Lipids**	**0.87**	DNA	0.49996
Cholesterol	0.08		
Lecithin	0.79	**Agarose**	**10.00**
**Recipe for regular food (RF).**			
**Ingredients**	**gram/Liter**		
Yeast	35.00		
Yellow cornmeal	80.00		
Dextrose	50.00		
10% p-Hydroxy-benzoic acid methyl ester			
in 95% ethanol (ml)	27.00		
Agar	9.00		

To assemble CDF, the appropriate amount of agarose and sugar (5X carbohydrate mix) are combined into a final volume of water (see File S1). This mixture is gently brought to a boil using a microwave to minimize evaporation. Once the solution cools to 65°C amino acid mixes (TD.10473 and TD.110036), basal mix (TD.10475), and lipid (100X stock) are added. The final solution is stirred without heating for an additional 5–10 minutes before aliquoting into vials. Plugged, boxed and wrapped vials are stable for 1 month at 4°C.

### Feeding Assays in Adult Flies

Newly eclosed adult flies were collected every 12 hours without CO_2_ anesthesia. 3 days later, 10 pairs of male and female flies were sorted into a fresh vial and aged for 3 additional days on regular food (RF; Table 1) before initiating the shift to experimental food. We began scoring values for survival, body weight, and egg-lay 12 hrs after the initial transfer onto experimental food. The 10 pairs of flies were transferred into fresh food vials of the appropriate type every other day during the course of an experiment.

### Measurements of Adult Body Weight and Egg-lay

Adult body weight was determined by performing two independent measurements of adult flies in a microcentrifuge tube using a precision balance then recording the average value. Average weight at each time point was normalized to initial average body weight. 12 hour egg-lay was determined every other day by counting the number of eggs present in a vial three times, recording the average value and then normalizing to the average number of living females present in the vial during consecutive time points. The accumulated egg-lay was calculated by summing average egg-lay values to a given time point. Flies used in both the survival and egg-laying studies were never anesthetized using CO_2_.

### Feeding Assays in Larvae

Newly eclosed adult flies were collected every 12 hours and grown on RF vials for 6 days before transferring into an egg-collecting bottle with grape juice plate. 24 hour egg-lays were collected on grape juice plates. Egg-lay plates were then inspected at two independent times over a 30 minute period to ensure all hatched larvae were completely removed. Individuals hatching within next 30 minute interval were then collected and 20–25 newly hatched 1^st^ instar larvae were transferred into experimental food vials to measure their development and viability. The time required for larval development was scored every 12 hours by counting the number of pupae present; each pupa was marked on the vial wall and followed to determine the time to eclosion.

### Trans-generational Feeding Assays

10 pairs of adult flies were collected and aged as described above. Flies were transferred into experimental vials at day 6 and into new vials 2 days later. For the second (and subsequent) generations, we collected 10–15 pairs of adult flies that eclosed within 3 days and transferred them into a fresh vial. Measurement of generation time was the same as described above.

### Temperature Shift experiments

10 pairs of adult flies were collected and aged as described above, then transferred into experimental vials and shifted to 18 or 29 degrees Celsius.

### Quantifying Effects of Dietary Macronutrients on Egg-lay

10 pairs of *w^1118^* flies were collected and aged for 6 days as described above and then transferred into new experimental food every day. Viability of adult flies and egg-lay was scored every 12 hours for 7 days. All CDF deficient media were compensated with remaining macronutrients, while maintaining proportional energetic contributions (see File S1). For example, amino acid deprivation CDF is compensated to 400 K-cal/L by adding extra sugar solution and fat mixture at a 4∶0.1 ratio.

### Statistics

Statistical analysis was performed using GraphPad Prism software Version 5.0d (GraphPad Software, Inc., CA, USA). Fisher’s exact tests were carried out using the online calculator from GraphPad Prism software homepage. Each statistical method used and corresponding p-values are listed in the Supplemental Tables. In all figure legends, *, **, *** indicate a p value <0.0500, <0.0100, <0.0010, respectively.

## Results and Discussion

**Figure 1:**
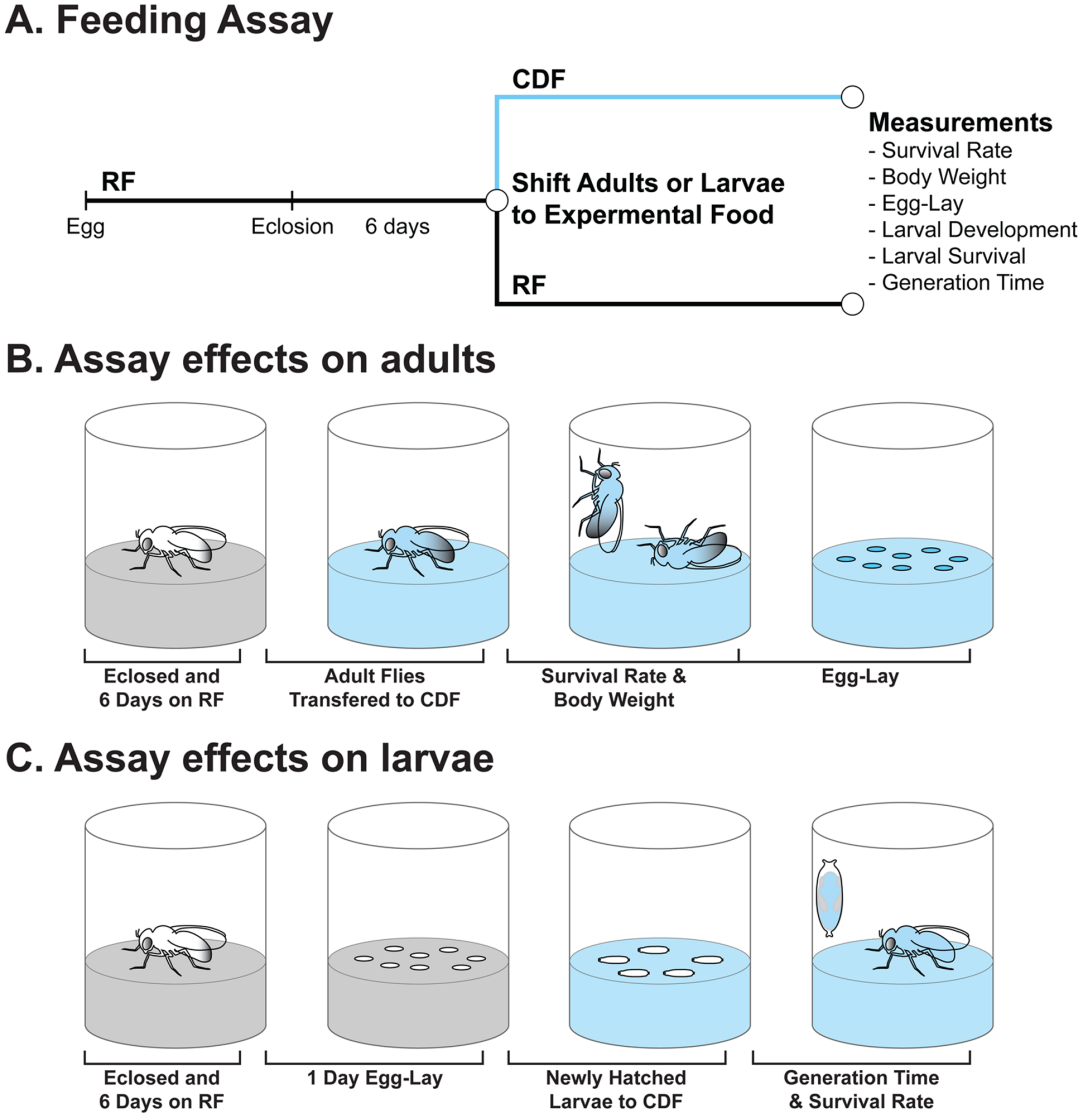
Experimental design. (A) General scheme for the feeding assays performed. Flies were grown and aged on regular food (RF) before shifting to chemically defined food (CDF). (B) Assays performed on adult flies. Body weight, survival, and egg-lay were measured after adult flies were shifted to chemically defined food. (C) Assays performed on developing flies. Larval development and survival were measured after newly hatched 1^st^ instar larvae were shifted onto chemically defined food.

In order to develop a chemically defined food (CDF) for *Drosophila* two general aspects of the media required optimization, dietary composition and caloric density (see Materials and Methods). Our goal was to synthesize a recipe that would functionally substitute for standard laboratory media. However, commonly used food recipes vary widely in their composition (e.g. http://flystocks.bio.indiana.edu/Fly_Work/media-recipes/media-recipes.htm). As a basis for comparison, we arbitrarily chose one standard *Drosophila* complex media, which we refer to here as regular food (RF; Table 1; see File S1). We combined a series of simple feeding assays with an iterative approach to empirically determine the effect of successive CDF formulations on broad indicators of organismal fitness including longevity, body weight and egg-laying ability, developmental time and trans-generation viability (Fig. 1). Table 1 summarizes the complete list of individual components in the final CDF recipe characterized in this study.

### CDF is Sufficient to Support the Culture of Adult *Drosophila*

#### Adult longevity

**Figure 2:**
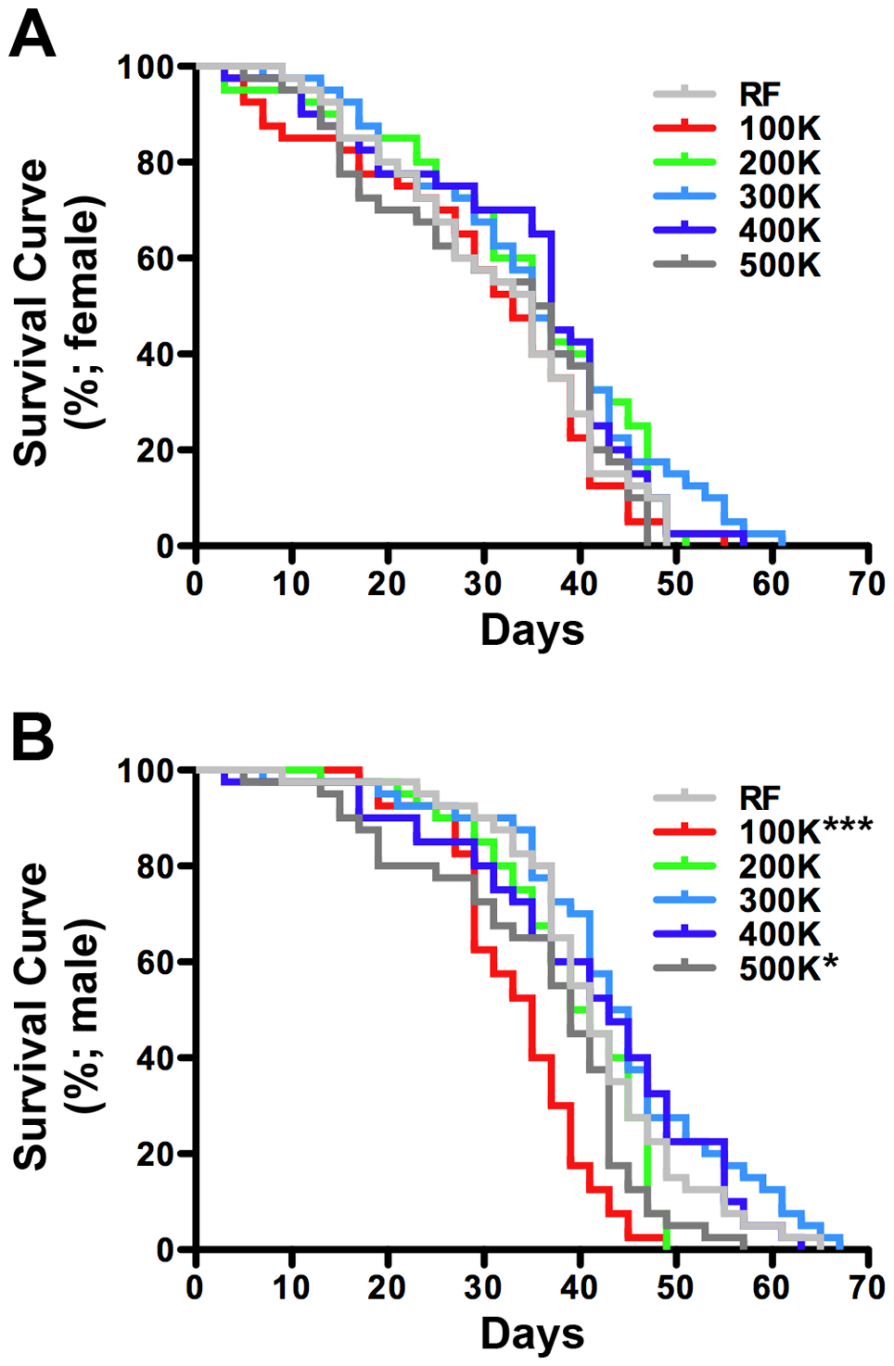
The effect of CDF on adult survival. (A) Survival of adult female flies cultured on chemically defined food as a function of caloric density. Comparison of all survival curves by long-rank (Mantel-Cox) test shows no statistical difference (n = 40; p≥0.1053 for all). (B) Survival of adult male flies cultured on chemically defined food as a function of caloric density. Paired comparison of survival curves between RF to other CDFs shows that the life span of males is only significantly reduced on 100 K-cal/L CDF (n = 40; p<0.0001) and slightly reduced on 500 K-cal/L CDF (n = 40, p = 0.0489). See Table S1 for additional details.

We first compared the viability of wild type (*white^1118^*) flies on both RF and CDF. On RF media, median survival values ranged from 35–41 days under our laboratory culture conditions (Table S1). Similar values were measured on CDF where median survival ranged from 33–44 days. Gender specific analysis showed that CDF in the range of 100–500 K-cal/L did not significantly affect life span of adult female flies when compared to RF (p>0.1053; Fig. 2A; Table S1). In contrast, adult males were found to be more sensitive to changes in caloric density, showing shorter life span on CDF^100K^ and CDF^500K^ (p<0.0001 and 0.0489 respectively) (Fig. 2B; Table S1). These results suggest that CDF formulated at a caloric density of between 200–400 K-cal/L is optimal to support the co-culture of adult male and female flies.

#### Adult weight

**Figure 3:**
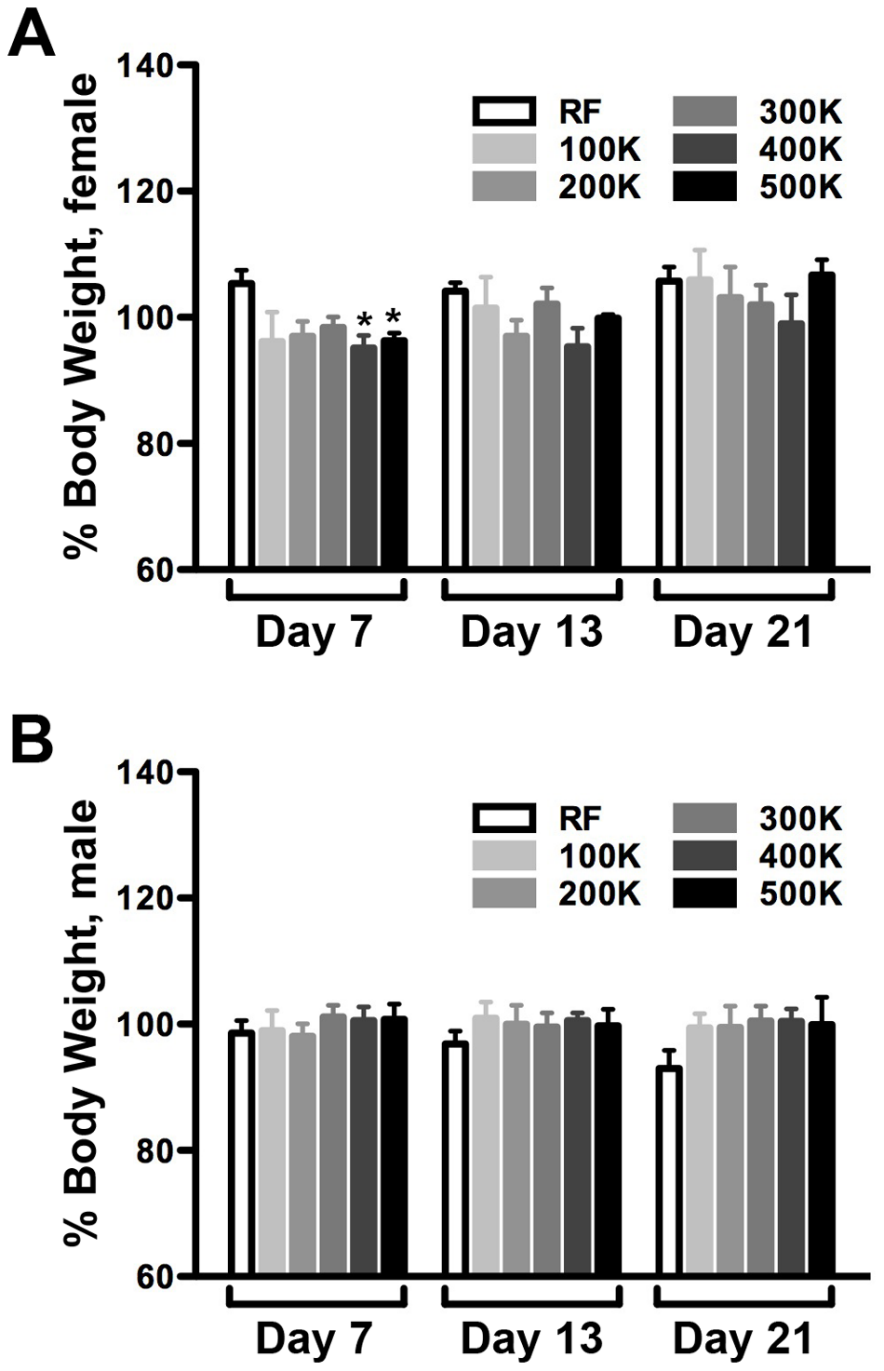
The effect of CDF on adult weight. (A) Average body weight of adult female flies cultured on chemically defined food as a function of caloric density. At day 7, only flies on 400 and 500 K-cal/L CDFs have less body weight than on RF (Mann Whitney test; n = 4, p = 0.0286 for both; see Table S2B for details). At day 13 and 21, the body weights of flies cultured on CDFs are not statistically different from flies cultured on RF. (B) Average body weight of adult male flies on chemically defined food as a function of caloric density. The body weights of flies cultured on all CDFs are not significantly different from flies cultured on RF at day 7, 13, and 21 (Mann Whitney test; n = 4, p≥0.1143 for all; see Table S2C for additional details).

We next determined the extent to which CDF diets affect adult body weight. On standard RF media, both male and female body weight was observed to gradually increase over time (Fig. S1A, Table S2A). We note that young flies exhibited little variation in measured weight, however this variation increased markedly in females with advancing age (Fig. S1A; Table S2A). This variation in weight in aged female flies may be related in part to dietary effects on egg-laying (see below). A similar trend was observed when we monitored changes in body weight in adult flies fed a CDF (Fig. S1B, C; Table S2B, C). When we compared the effect of RF and CDF on the weight of young flies at defined time points, no significant differences were detected, with the exception of 400 K-cal/L and 500 K-cal/L diets on day 7 females (Fig. 3A, B; Table S2B, C). Thus, CDF diets were not associated with significant changes in overall adult body weight compared to standard RF media.

#### Female egg-lay

**Figure 4:**
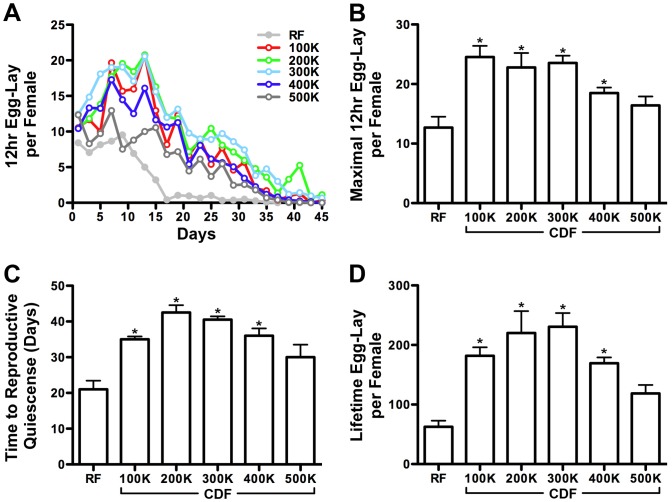
The effect of CDF on adult female egg-laying. Chemically defined food extends the egg-laying ability of adult female flies. (A) Average egg-lay in 12 hours of adult female flies on chemically defined food as a function of caloric density. Females fed on CDF show an increase in both maximal egg-lay and reproductive lifespan (n = 4). B) Maximal 12-hour egg-lay on chemically defined food. CDF^100K–400K^ enhances the maximal egg-laying ability compared to RF (Mann Whitney test; n = 4, p = 0.2454 for CDF^500K^ and 0.0286 for others). (C) Number of days a female is capable of producing more than one egg per day. CDF^100K–400K^ extends the reproductive lifespan compared to RF (Mann Whitney test; n = 4, p = 0.1441 for CDF^500K^ and ≤0.0294 for others). (D) Total lifetime egg-lay per female on chemically defined food. Females lay more eggs on CDF^100K–400K^ than on RF (Mann Whitney test; n = 4, p = 0.0571 for CDF^500K^ and 0.0286 for others). See Table S3 for additional details.

Finally, we wished to determine if CDF diets affect female egg-laying ability. To quantify this effect, we first scored the number of eggs laid per female over the course of adult life (Fig. 4A; Table S3). This analysis showed that females fed a RF diet lay a maximum of 12±1.8 eggs in 12 hours, whereas females fed a CDF have a maximum egg-lay as high as 24.6±1.9 (Fig. 4B; Table S3). We next examined whether CDF could influence the female reproductive life span. To quantify this phenotype we calculated the time to reproductive quiescence defined as the number of days a female can lay more than a single egg per day. Females fed a RF diet remain reproductively active period for 21.0±2.4 days (Fig. 4C). Females fed a CDF diet showed an increase in reproductive longevity at all caloric densities tested with averages of 35.0±0.8, 42.5±2.1, 40.5±1.0, 36.0±2.1, 30.0±3.5 days on CDF^100K^, CDF^200K^, CDF^300K^, CDF^400K^, CDF^500K^ respectively (Fig. 4C; Table S3). Finally, to calculate total lifetime egg-lay we summed each independent 12-hour count over the duration of the experiment. Females fed a RF diet lay a lifetime average of 62.7±10.1 eggs (Fig. 4D). In contrast, females fed a CDF diet showed an increase in reproductive activity at all caloric densities tested with lifetime averages of 182.0±14.1, 220.0±36.9, 230.7±23.1; 169.4±9.8; 118.6±14.3 eggs on CDF^100K^, CDF^200K^, CDF^300K^, CDF^400K^, CDF^500K^ respectively (Fig. 4D; Table S3). Thus, CDF diets were associated with an increase in the rate of egg-lay, reproductive longevity and total reproductive capacity of females.

In summary, the effects of a chemically defined food were compared to a standard *Drosophila* media. Gross measures of adult homeostasis were similar on RF and CDF, although in some cases male and female measurements diverged, suggesting distinct dietary requirements. Finally, this analysis directly demonstrates that caloric density affects measures of adult longevity, body weight and egg-lay.

### CDF is Sufficient to Support the Culture of Developing *Drosophila*

**Figure 5:**
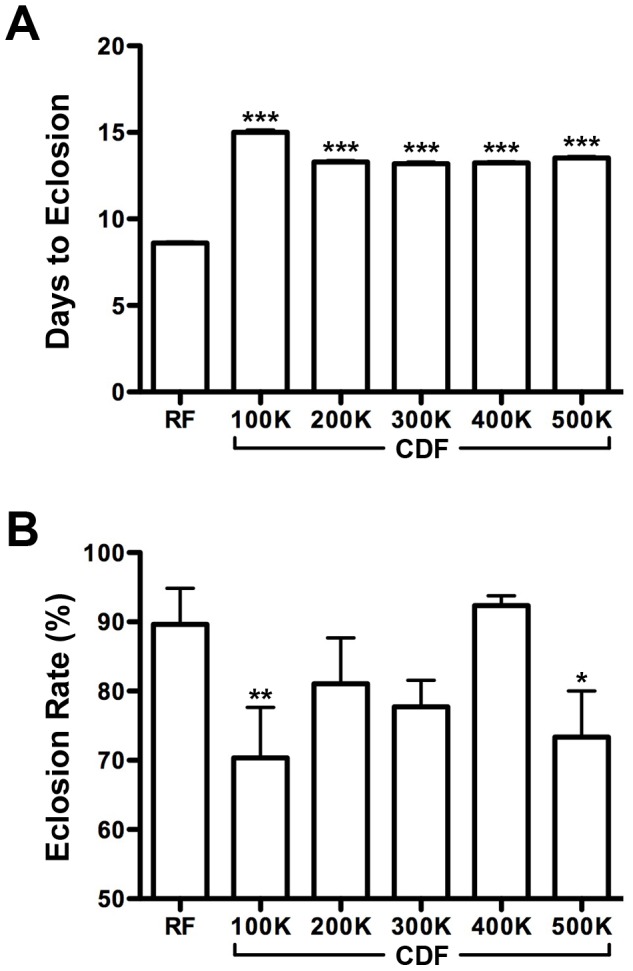
The effect of CDF on larval development and survival. (A) Days required for first instar larvae to eclose on chemically defined food. Larvae grown on CDFs show statistically significant developmental delay (Mann Whitney test; n ≥65, p<0.0001 for all; see Table S4A for details). (B) Eclosion rates for first instar larvae cultured on chemically defined food. Larvae on CDF^200K–400K^ show no statistical difference in survival compared to RF (one-tailed Fisher’s exact test; n ≥65, p≥0.0544 for all; see Table S4B for details), but lower survival is observed on CDF^100K^ and CDF^500K^ (p = 0.0025 and 0.0202 respectively).

To determine if CDF was sufficient to support early growth of *Drosophila*, we compared the developmental rate and survival of larvae reared on either RF or CDF. Embryos were collected from adults cultured on RF (Fig. 1A, C). Following hatching, larvae were either maintained on RF or transferred to a CDF. We first determined if CDF affects the time necessary to complete larval development by scoring the number of larvae that reached pupation and/or eclosion every 12 hours, following a timed egg-lay. These studies showed that without exception CDF diets were associated with a significant developmental delay (Fig. 5A). The average time to eclosion was 8.6 days on RF, while time to eclosion on CDF ranged from 13.2–15 days depending on the caloric density of the media (Table S4A). Temporal analysis revealed that most, if not all, of this effect occurred during the larval stages of development (Fig. S2A).

We then determined if the observed developmental delay was associated with lethality during development. To assess this, we measured the survival rate of embryos from hatching to eclosion on both RF and CDF media. The average survival rate on RF was 89.7±5.2 percent, whereas percent survival on CDF ranged from 70.3±7.3–92.3±1.5 depending on the caloric density of the media (Fig. 5B). Survival on CDF^200–400K^ trended lower but did not significantly differ from survival on RF (Table S4B). Temporal analysis indicated that for those diets associated with significantly lower survival rates (i.e. CDF^100K^ and CDF^500K^) death occurred largely during the pupal period (Fig. S2B). Taken together these studies indicate that CDF can also support *Drosophila* development. While CDF is associated with a significant developmental delay, a caloric density 400 K-cal/L was associated with the shortest developmental delay and lowest lethality.

### CDF is Sufficient to Support Long-term Culture of *Drosophila*

**Figure 6:**
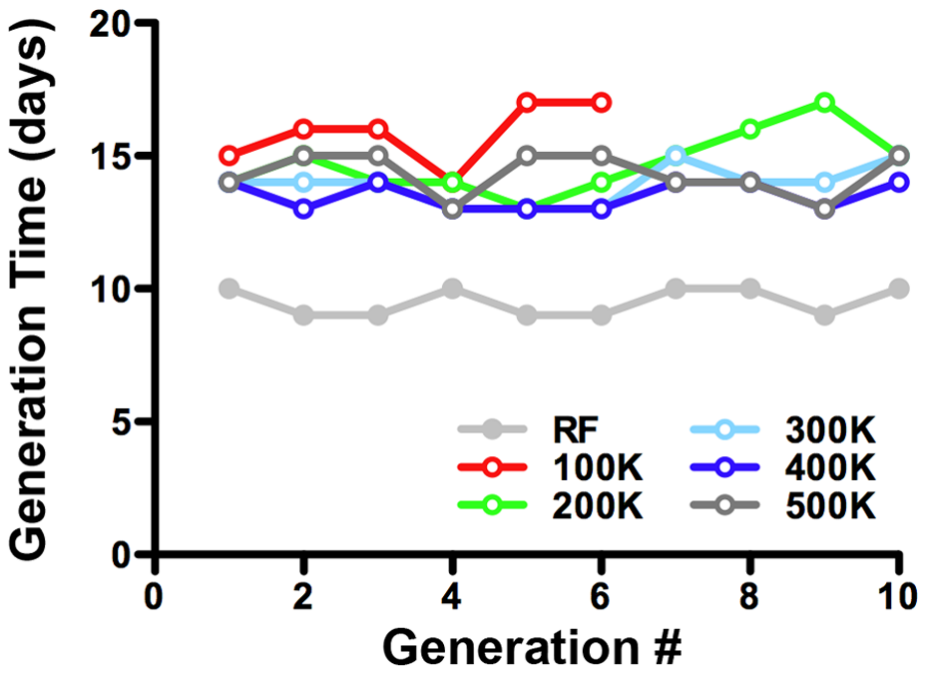
CDF is sufficient to support long-term culture of *Drosophila* strains. Generation number as a function of caloric density. CDFs over 200 K-cal/L successfully support trans-generational propagation of *Drosophila* strains.

A stringent test of a CDF is the ability to support trans-generational propagation of individual cultures, as incomplete diets ultimately lead to a lack of viability on deficient media. To test the ability of CDF to support long-term culture we monitored both the number of successive generations and generation times of cultures grown on either RF or CDF (Fig. 6). Our studies show that CDF was sufficient to support trans-generational growth for 10 successive generations. This was most clearly the case for CDF formulated at higher caloric densities (i.e. 300–500 K-cal/L); CDF at 100 K-cal/L ultimately failed to support growth. Generation times for flies cultured on a particular diet were not observed to change from one generation to the next. As described above most of the developmental delay observed in a given generation is attributable to effects on larval development. Subsequent to these studies, cultures have been continuously propagated for up to 30 generations (Table 2), although generation times were not quantified after the 10^th^ generation. We also noted that CDF is capable of supporting culture growth at common experimental conditions of both 18 and 29 degrees Celsius (Table 2). Taken together, these experiments demonstrate that CDF is sufficient to support long-term culture of *Drosophila* strains under experimentally relevant conditions. Table 2 summarizes our observations concerning the culture of *Drosophila* on RF and CDF of different caloric densities.

**Table 2:** Summary: chemically defined food versus regular food.

Parameters	CDF^100K^	CDF^200K^	CDF^300K^	CDF^400K^	CDF^500K^
**Female**					
Survival	n.s.	n.s.	n.s.	n.s.	n.s.
Body weight	n.s.	n.s.	n.s.	n.s.	n.s.
Lifetime egg-lay	2.9 fold increase	3.5 fold increase	3.7 fold increase	2.7 fold increase	n.s.
**Male**					
Survival	6 days shorter	n.s.	n.s.	n.s.	2 days shorter
Body weight	n.s.	n.s.	n.s.	n.s.	n.s.
**Larvae**					
Survival	19.3% decrease	n.s.	n.s.	n.s.	16.3% decrease
Growth	6.05 days delayed	4.25 days delayed	4.25 days delayed	4.14 days delayed	4.31 days delayed
Pupation period	0.43 days delayed	0.38 days delayed	0.47 days delayed	0.42 days delayed	0.52 days delayed
**Transgeneration**					
Generation time	5.6 days delayed	5.2 days delayed	3.6 days delayed	2.6 days delayed	2.8 days delayed
Growth @ 29°C	+	+	+	+	+
Growth @ 18°C	− ^a^	+/− ^a^	+ ^a^	+	+
Generation #	6	>30	>30	>30	>30

n.s.: not statistically significant; +: vigorous culture growth; +/−: poor culture growth; -: fail to support culture; ^a^ vials often have fungi/bacterial growth.

### CDF can be used to Distinguish Nutritional Requirements from Caloric Requirements in *Drosophila*

We wished to determine the effect of individual macronutrients (amino acids, carbohydrates and fat) on developmental and homeostatic processes, independent of any potential effects of altered caloric density. Of the caloric densities tested in the experiments described above, CDF formulated at 400 K-cal/L consistently led to measures that were most similar to RF media over a rage of different assays. Thus we selected CDF at 400 K-cal/L for use in these “drop-out” studies. Holding caloric density constant, we examined the effect of deficits in each of the three macronutrients in our assays of adult survival, female egg-lay and larval development. Note that in the “drop-out” studies described here, caloric density that would have been lost from the diet by eliminating amino acids (for example) is compensated by augmenting both carbohydrates and fat, while holding the overall proportions of remaining macronutrients constant (see Materials and Methods; File S1).

**Figure 7:**
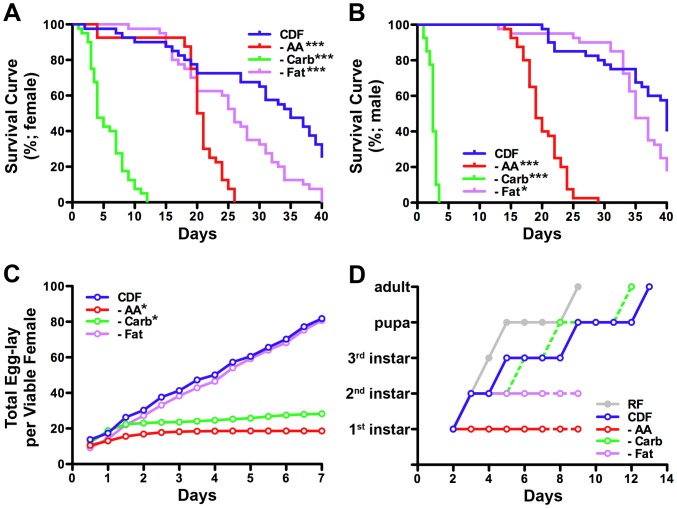
The effects of macronutrient deficiency on adult survival, female egg-lay and larval development. (A) Survival of adult female flies on chemically defined food (CDF) formulated at a caloric density of 400 K-cal/L and lacking either amino acids (AA), carbohydrates (Carb) or fats. Comparison of survival curves among all groups by long-rank (Mantel-Cox) test shows that life span is significantly reduced under each of the deprivation conditions (n = 40, p≤0.0002 for all; see Table S5A for details). (B) Survival of adult male flies on chemically defined food formulated at a caloric density of 400 K-cal/L and lacking either amino acids, carbohydrates or fats. Comparison of all survival curves in male flies shows the life span of males is significantly reduced on tested deprivation conditions (n = 40, p≤0.0241 for all; see Table S5A for details). (C) Total egg-lay per viable female on chemically defined food lacking either amino acids, carbohydrates or fats. Females lay fewer eggs on CDF lacking either amino acids or carbohydrates (Mann Whitney test; n = 4, p = 0.0286 for both), but not fat (p = 0.7715; see Table S5B for additional details). (D) Larval development on chemically defined food lacking either amino acids, carbohydrates or fats. Larvae fed on CDF lacking amino acids show growth arrest at 1^st^ instar stage. Larvae fed on CDF lacking carbohydrates show high lethality in 2^nd^ instar stage, but escapers can progress to adulthood (see text). Larvae fed on fat deprived CDF show growth arrest at 2^nd^ instar stage. CDF: CDF^400K^; - AA: amino acid deprived CDF^400K^; - Carb: carbohydrate deprived CDF^400K^: - Fat: fat deprived CDF^400K^; Dashed line indicates lethality.

We first compared the effects of serially eliminating each macronutrient from CDF on adult survival. In both males and females, dietary amino acids, carbohydrates, and fats were all found to be required for adult survival (Fig. 7A, B; Table S5A). The median survival of adult females deprived of amino acids (CDF^400K−AA^), carbohydrates (CDF^400K−Carb^), or fat (CDF^400K−Fat^) is 20.5, 4.0, and 26.0 days respectively. In adult males, median survival was, 19.0, 2.5, and 35.0 days on CDF^400K−AA^, CDF^400K−Carb^, and CDF^400K−Fat^. These studies demonstrate that under experimental conditions where caloric density is held constant (i.e. 400 K-cal/L), dietary carbohydrates play the most important role in adult longevity, followed by amino acids and then fat. Although adult male flies are more sensitive to dietary carbohydrate deprivation than females, they are less sensitive to fat deprivation. Thus, nutritional requirements for the survival of adult flies differ between genders.

We next compared the effects of serially eliminating each macronutrient from CDF on female egg-laying ability. In these studies we scored the number of eggs laid per female every 12 hours for 7 consecutive days. We found that female egg-lay was differentially sensitive to macronutrient deprivation (Fig. 7C; Table S5B). For example, total egg-lay of female flies fed either CDF or CDF lacking fat did not significantly differ (81.8±3.6, 80.7±5.1, respectively). However, females fed CDF lacking either amino acids or carbohydrates produced significantly fewer eggs (18.7±2.5, 28.3±3.0, respectively). Thus, under experimental conditions in which caloric density is held constant (i.e. 400 K-cal/L), both amino acids and carbohydrates are necessary for maintaining female egg-laying ability, while fat is dispensable.

Finally we tested the effects of serially eliminating macronutrients from CDF on developmental progression. In these studies we scored the time required to progress through larval and pupal stages. We found that post-embryonic development was differentially sensitive to the type of macronutrient deprivation (Fig. 7D). Not surprisingly, significant lethality and developmental delay was found to be associated with macronutrient deficits. For example, only a small fraction (less than 2%) of larvae grown on CDF lacking carbohydrates grew to adulthood, and were delayed in their development. Even more extreme requirements were observed with deficits in amino acids and fat. Larvae grown on either amino acid or fat deprived CDF showed developmental arrest and died 7 days after egg-lay. Thus, under experimental conditions in which caloric density is held constant (i.e. 400 K-cal/L) carbohydrates, amino acids and fat are all necessary for larval development.

In summary, we have developed a chemically defined food (CDF) for the analysis of macro- and micronutrients in *Drosophila*. We have characterized the effects of this diet on both developmental and homeostatic processes and show that CDF can functionally substitute for standard media in a number of independent assays. While CDF is sufficient to support the long-term culture of *Drosophila* strains, it is associated with a significant delay in larval development. Replacement of dietary protein with amino acid mixes has previously been shown to prolong larval development and in some insects disrupt osmotic balance during development [37], [38]. Therefore, additional modifications are necessary to optimize CDF for larval growth. Importantly, we demonstrate that CDF allows the effects of macronutrient and caloric density requirements to be distinguished experimentally. The CDF recipe described here should, in principle, permit the systematic experimental manipulation of individual nutrients within the diet (i.e. single essential amino acids). Similarly, this recipe can easily be used to test the effects of augmenting macro- or micronutrient composition or overall caloric density in the range above 500 K-cal/L. In *Drosophila*, methods to manipulate gene function at the single cell level can combine powerfully with the ability to manipulate specific dietary components leading to new insights into the way in which nutrient availability affects developmental, homeostatic and disease processes.

## Supporting Information

Figure S1 **Effect of CDF on adult weight.** (A) Average body weight of adult flies cultured on regular food (RF) as a function of age. Females gain 5.7±2.2 (Mean±SE), 14.9±3.9, 21.2±7.8% of body weight at day 21, 27, 35 respectively (Mann Whitney test; n = 4 except at day 35; p≥0.1288 for all; see Table S2A for additional details). Males lose 7.0±2.8 and 1.3±2.0% of body weight by day 21 (n = 4, p = 0.0289) and day 27 respectively, then gain 2.5±5.9% of body weight by day 35 (n = 4, p = 0.4754). (B) Average body weight of adult female flies cultured on chemically defined food (CDF) as a function of caloric density. In the first week on CDF, females first lose about 10% of their initial body weight which is recovered by day 5. Females gain 21.2±7.8, 10.1±5.7, 4.7±2.6, 13.3±2.0, 8.1±4.2 and 12.4±5.1% of body weight after 35 days on RF, CDF^100K^, CDF^200K^, CDF^300K^, CDF^400K^ and CDF^500K^ respectively (Mann Whitney test; n = 4, p≥0.1143 for all; see Table S2B for details). Females on CDF show a similar trend of increasing body weight as they age on RF (Friedman test; n = 4, p≤0.0006 for all; see Table S2D for details). (C) Average body weight of adult male flies cultured on chemically defined food as a function of caloric density. Males gain 2.5±5.9, 7.8±3.4, 10.2±1.2, 14.0±2.0, 11.2±3.2 and 12.7±5.6% of body weight changes after 35 days on RF, CDF^100K^, CDF^200K^, CDF^300K^, CDF^400K^ and CDF^500K^ respectively (Mann Whitney test; n = 4, p≥0.200 for all; see Table S2C for details). Male flies on CDFs show an increasing trend in body weight compared to males aged on RF (Friedman test; n = 4, p≤0.0116 for all; see Table S2D for details).
(TIF)

Figure S2 **Effect of CDF on larval development and survival.** (A) Days required for larvae to complete different stages of development when cultured on chemically defined food. All larvae grown on CDFs show a statistically significant developmental delay (Mann Whitney test; n ≥65, p<0.0001 for all; see Table S4A for details). (B) Survival rates for larvae cultured on chemically defined food by stage. Larvae cultured on CDF^200K–400K^ show no statistical difference in survival compared to RF (one-tailed Fisher’s exact test; n >65, p≥0.0544 for all; see Table S4B for additional details); significant differences in survival are observed on CDF^100K^ and CDF^500K^ (p = 0.0025 and 0.0202 respectively).
(TIF)

Table S1 **Longevity of adult flies on CDF.**
(PDF)

Table S2 **Body weight of adult flies on RF or CDF.**
(PDF)

Table S3 **Effect of CDF on egg-lay.**
(PDF)

Table S4 **Larval development and survival on CDF.**
(PDF)

Table S5 **Effect of macro-nutrient deprivation on adult longevity and egg-lay.**
(PDF)

File S1 **Summary of food recipes used in this study.**
(XLSX)
