# Supplementary figures and images for: Development and Characterization of a Chemically Defined Food for Drosophila
Source: PLoS One. 2013 Jul 2;8(7):e67308. doi: 10.1371/journal.pone.0067308 (PMC3699577; doi:10.1371/journal.pone.0067308)

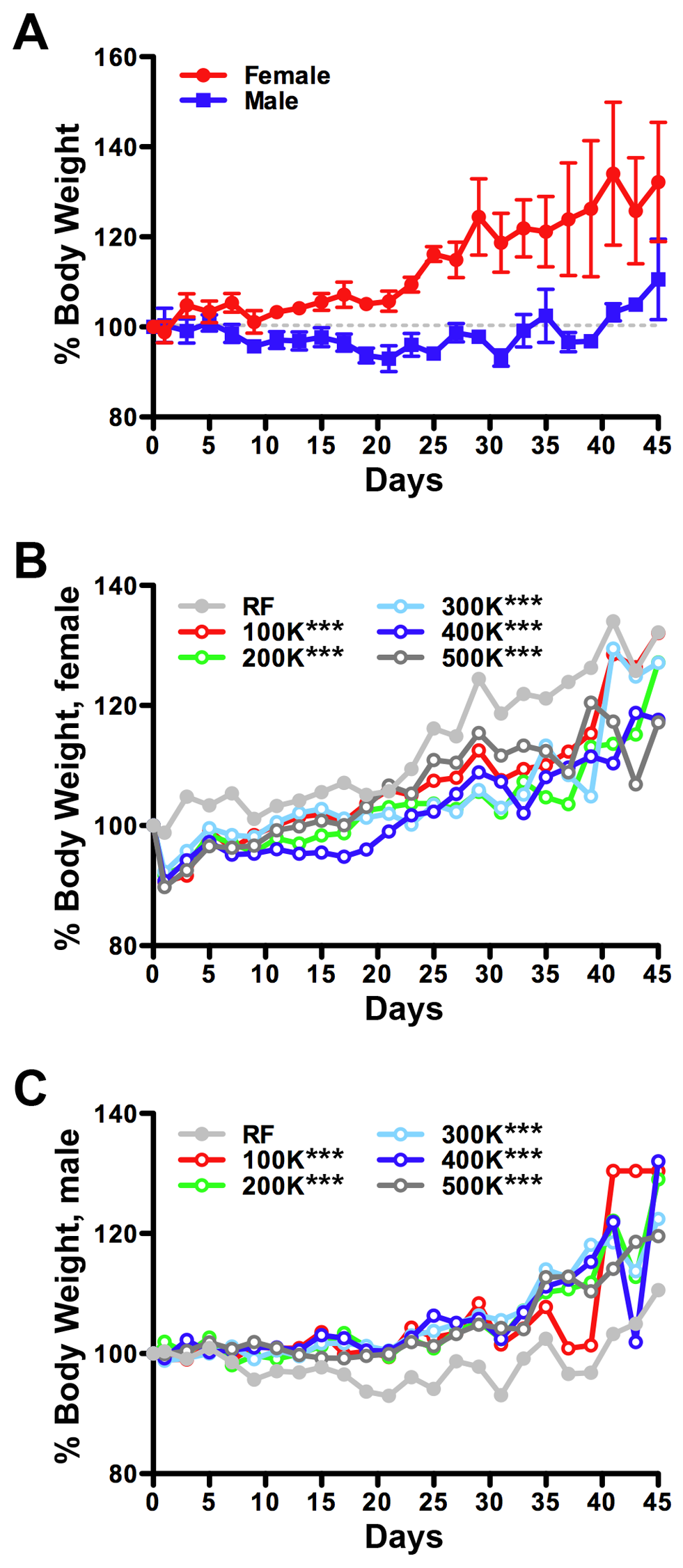

Supplement: Figure S1 — Effect of CDF on adult weight. (A) Average body weight of adult flies cultured on regular food (RF) as a function of age. Females gain 5.7±2.2 (Mean±SE), 14.9±3.9, 21.2±7.8% of body weight at day 21, 27, 35 respectively (Mann Whitney test; n = 4 except at day 35; p≥0.1288 for all; see Table S2A for additional details). Males lose 7.0±2.8 and 1.3±2.0% of body weight by day 21 (n = 4, p = 0.0289) and day 27 respectively, then gain 2.5±5.9% of body weight by day 35 (n = 4, p = 0.4754). (B) Average body weight of adult female flies cultured on chemically defined food (CDF) as a function of caloric density. In the first week on CDF, females first lose about 10% of their initial body weight which is recovered by day 5. Females gain 21.2±7.8, 10.1±5.7, 4.7±2.6, 13.3±2.0, 8.1±4.2 and 12.4±5.1% of body weight after 35 days on RF, CDF100K, CDF200K, CDF300K, CDF400K and CDF500K respectively (Mann Whitney test; n = 4, p≥0.1143 for all; see Table S2B for details). Females on CDF show a similar trend of increasing body weight as they age on RF (Friedman test; n = 4, p≤0.0006 for all; see Table S2D for details). (C) Average body weight of adult male flies cultured on chemically defined food as a function of caloric density. Males gain 2.5±5.9, 7.8±3.4, 10.2±1.2, 14.0±2.0, 11.2±3.2 and 12.7±5.6% of body weight changes after 35 days on RF, CDF100K, CDF200K, CDF300K, CDF400K and CDF500K respectively (Mann Whitney test; n = 4, p≥0.200 for all; see Table S2C for details). Male flies on CDFs show an increasing trend in body weight compared to males aged on RF (Friedman test; n = 4, p≤0.0116 for all; see Table S2D for details). (TIF) [file pone.0067308.s001.tif]

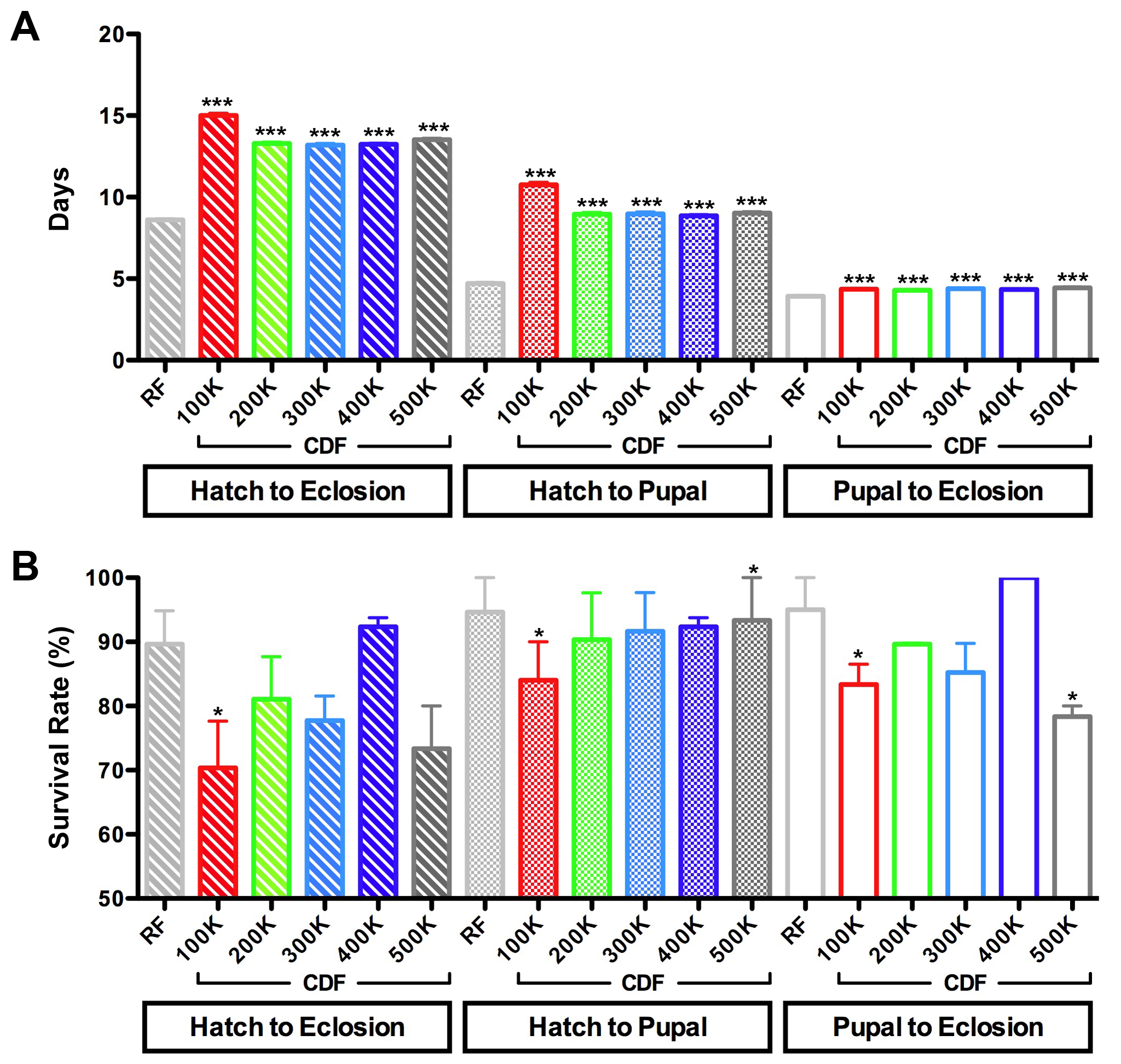

Supplement: Figure S2 — Effect of CDF on larval development and survival. (A) Days required for larvae to complete different stages of development when cultured on chemically defined food. All larvae grown on CDFs show a statistically significant developmental delay (Mann Whitney test; n ≥65, p<0.0001 for all; see Table S4A for details). (B) Survival rates for larvae cultured on chemically defined food by stage. Larvae cultured on CDF200K–400K show no statistical difference in survival compared to RF (one-tailed Fisher’s exact test; n >65, p≥0.0544 for all; see Table S4B for additional details); significant differences in survival are observed on CDF100K and CDF500K (p = 0.0025 and 0.0202 respectively). (TIF) [file pone.0067308.s002.tif]
