# Supplementary material for: Development and Characterization of a Chemically Defined Food for Drosophila
Source: PLoS One. 2013 Jul 2;8(7):e67308. doi: 10.1371/journal.pone.0067308 (PMC3699577; doi:10.1371/journal.pone.0067308)
Supplement: Table S1 — Longevity of adult flies on CDF. (PDF) [file pone.0067308.s003.pdf]

Supplemental Table S1. Longevity of adult flies on CDF (n = 40, 4 replicates, 10 pairs of flies for each replicate)**.** Statistically significant p values are labeled with bold text.

		RF	CDF^{100K}	CDF^{200K}	CDF^{300K}	CDF^{400K}	CDF^{500K}
Female median survival	Days	35	33	36	35	37	36
Female median survival	p value for Mantel-Cox test to RF		0.6796	0.2577	0.1053	0.2425	0.7777
Male median survival	Days	41	35	40	44	43	39
Male median survival	p value for Mantel-Cox test to RF		**< 0.0001**	0.1789	0.1600	0.6631	**0.0489**
