# Supplementary material for: Development and Characterization of a Chemically Defined Food for Drosophila
Source: PLoS One. 2013 Jul 2;8(7):e67308. doi: 10.1371/journal.pone.0067308 (PMC3699577; doi:10.1371/journal.pone.0067308)
Supplement: Table S2 — Body weight of adult flies on RF or CDF. (PDF) [file pone.0067308.s004.pdf]

Supplemental Table S2A. Body weight of adult flies on RF (4 replicates, 10 pairs of flies for each replicate; ‡ 3 replicates; normalized to day 0)**.** Statistically significant p values are labeled with bold text.

		Day 0	Day 7	Day 13	Day 21	Day 27	Day 35
Female	Mean	100	105.4	104.2	105.7	114.9	121.2‡
Female	Std. Error	0.0	2.1	1.3	2.2	3.9	7.8‡
Female	p value for Mann Whitney test to Day 0		0.0821	0.4930	0.4952	0.1288	0.2110‡
Male	Mean	100.0	98.6	96.9	93.0	98.7	102.5
Male	Std. Error	0.0	2.0	2.0	2.8	2.0	5.9
Male	p value for Mann Whitney test to Day 0		0.5308	0.2408	**0.0289**	0.1057	0.4754

Supplemental Table S2B. Body weight of adult female flies on CDF (4 replicates, 10 pairs of flies for each replicate; normalized to day 0)**.** Statistically significant p values are labeled with bold text.

		RF	CDF^{100K}	CDF^{200K}	CDF^{300K}	CDF^{400K}	CDF^{500K}
Female (day 7)	Mean	105.4	96.2	97.0	98.4	95.2	96.3
Female (day 7)	Std. Error	2.1	4.7	2.3	1.6	1.9	1.2
Female (day 7)	p value for Mann Whitney test to RF		0.3429	0.0571	0.0571	**0.0286**	**0.0286**
Female (day13)	Mean	104.2	101.5	97.0	102.1	95.3	99.9
Female (day13)	Std. Error	1.3	4.9	2.6	2.5	3.0	0.6
Female (day13)	p value for Mann Whitney test to RF		0.8857	0.0814	0.6857	0.0571	0.0571
Female (day21)	Mean	105.7	106.0	103.1	102.0	99.0	106.7
Female (day21)	Std. Error	2.2	4.7	4.9	3.1	4.6	2.5
Female (day21)	p value for Mann Whitney test to RF		0.8857	0.4857	0.3836	0.4857	1.0000
Female (day27)	Mean	114.9	108.0	102.8	102.3	105.3	110.5
Female (day27)	Std. Error	3.9	2.0	3.6	2.6	4.2	5.5
Female (day27)	p value for Mann Whitney test to RF		0.3429	0.0571	0.0571	0.1143	0.4857
Female (day35)	Mean	121.2‡	110.1	104.7	113.3	108.1	112.4
Female (day35)	Std. Error	7.8‡	5.7	2.6	2.0	4.2	5.1
Female (day35)	p value for Mann Whitney test to RF		0.2286	0.1143	0.4000	0.2286	0.4000

Supplemental Table S2C. Body weight of adult male flies on CDF (4 replicates, 10 pairs of flies for each replicate; normalized to day 0)**.** Statistically significant p values are labeled with bold text.

		RF	CDF^{100K}	CDF^{200K}	CDF^{300K}	CDF^{400K}	CDF^{500K}
Male (day 7)	Mean	98.6	99.0	98.1	101.2	100.6	100.8
Male (day 7)	Std. Error	2.0	3.2	2.0	1.8	2.2	2.4
Male (day 7)	p value for Mann Whitney test to RF		0.8857	1.0000	0.3429	0.4857	0.4857
Male (day13)	Mean	96.9	101.0	99.9	99.6	100.6	99.8
Male (day13)	Std. Error	2.0	2.6	3.0	2.2	1.2	2.6
Male (day13)	p value for Mann Whitney test to RF		0.2000	0.6857	0.2454	0.2000	0.2000
Male (day21)	Mean	93.0	99.5	99.5	100.5	100.5	100.0
Male (day21)	Std. Error	2.8	2.2	3.4	2.4	2.0	4.3
Male (day21)	p value for Mann Whitney test to RF		0.2000	0.1465	0.1143	0.1143	0.1143
Male (day27)	Mean	98.7	103.5	103.7	104.9	105.1	103.3
Male (day27)	Std. Error	2.0	1.2	2.9	1.8	2.5	3.9
Male (day27)	p value for Mann Whitney test to RF		0.1143	0.3094	0.1143	0.1143	0.4857
Male (day35)	Mean	102.5	107.8	110.2	114.0	111.2	112.7
Male (day35)	Std. Error	5.9	3.4	1.2	2.0	3.2	5.6
Male (day35)	p value for Mann Whitney test to RF		0.6857	0.3429	0.2000	0.3429	0.3429

Supplemental Table S2D. Trends in body weight of adult flies fed on CDF (4 replicates, 10 pairs of flies for each replicate)**.** Statistically significant p values are labeled with bold text. Day 33 is the last data point eligible for Friedman test in female flies.

		CDF^{100K}	CDF^{200K}	CDF^{300K}	CDF^{400K}	CDF^{500K}
Female (day 1-21)	p value for Friedman test to RF	**0.0006**	**< 0.0001**	**< 0.0001**	**< 0.0001**	**0.0006**
Female (day 1-33)	p value for Friedman test to RF	**< 0.0001**	**< 0.0001**	**< 0.0001**	**< 0.0001**	**< 0.0001**
Male (day 1-21)	p value for Friedman test to RF	**0.0116**	**0.0006**	**0.0116**	**0.0116**	**0.0006**
Male (day 1-45)	p value for Friedman test to RF	**< 0.0001**	**< 0.0001**	**< 0.0001**	**< 0.0001**	**< 0.0001**
