# Supplementary material for: Development and Characterization of a Chemically Defined Food for Drosophila
Source: PLoS One. 2013 Jul 2;8(7):e67308. doi: 10.1371/journal.pone.0067308 (PMC3699577; doi:10.1371/journal.pone.0067308)
Supplement: Table S3 — Effect of CDF on egg-lay. (PDF) [file pone.0067308.s005.pdf]

Supplemental Table S3. Effect of CDF on egg-lay (4 replicates, 10 pairs of flies for each replicate)**.** Statistically significant p values are labeled with bold text.

		RF	CDF^{100K}	CDF^{200K}	CDF^{300K}	CDF^{400K}	CDF^{500K}
Maximal 12 hr egg-lay	Mean	12.7	24.6	22.8	23.6	18.5	16.4
Maximal 12 hr egg-lay	Std. Error	1.8	1.9	2.4	1.2	0.9	1.5
Maximal 12 hr egg-lay	p value for Mann Whitney test to RF		**0.0286**	**0.0286**	**0.0286**	**0.0286**	0.2454
Days of maximal egg-lay	Mean	6.5	10.5	11.0	12.0	12.5	5.0
Days of maximal egg-lay	Std. Error	1.9	1.5	1.4	1.7	2.1	1.4
Days of maximal egg-lay	p value for Mann Whitney test to RF		0.2308	0.1416	0.1416	0.1416	0.3688
Time to Reproductive Quiescence (days)	Mean	21.0	35.0	42.5	40.5	36.0	30.0
Time to Reproductive Quiescence (days)	Std. Error	2.4	0.8	2.1	1.0	2.1	3.5
Time to Reproductive Quiescence (Days)	p value for Mann Whitney test to RF		**0.0284**	**0.0284**	**0.0284**	**0.0294**	0.1441
Lifetime egg-lay	Mean	62.7	182.0	220.0	230.7	169.4	118.6
Lifetime egg-lay	Std. Error	10.1	14.1	36.9	23.1	9.8	14.3
Lifetime egg-lay	p value for Mann Whitney test to RF		**0.0286**	**0.0286**	**0.0286**	**0.0286**	0.0571
