# Supplementary material for: Development and Characterization of a Chemically Defined Food for Drosophila
Source: PLoS One. 2013 Jul 2;8(7):e67308. doi: 10.1371/journal.pone.0067308 (PMC3699577; doi:10.1371/journal.pone.0067308)
Supplement: Table S4 — Larval development and survival on CDF. (PDF) [file pone.0067308.s006.pdf]

Supplemental Table S4A. Larval development on CDF (n ≥ 65, 3 replicates, 20-25 larvae for each replicate)**.** Statistically significant p values are labeled with bold text.

		RF	CDF^{100K}	CDF^{200K}	CDF^{300K}	CDF^{400K}	CDF^{500K}
Days (Hatch to Eclosion)	Mean	8.61	15.00	13.29	13.19	13.23	13.52
Days (Hatch to Eclosion)	Std. Error	0.06	0.15	0.09	0.11	0.08	0.07
Days (Hatch to Eclosion)	p value for Mann Whitney test to RF		**< 0.0001**	**< 0.0001**	**< 0.0001**	**< 0.0001**	**< 0.0001**
Days (Hatch to Pupal)	Mean	4.71	10.76	8.97	8.97	8.85	9.03
Days (Hatch to Pupal)	Std. Error	0.06	0.15	0.09	0.11	0.08	4.44
Days (Hatch to Pupal)	p value for Mann Whitney test to RF		**< 0.0001**	**< 0.0001**	**< 0.0001**	**< 0.0001**	**< 0.0001**
Days (Pupal to Eclosion)	Mean	3.92	4.35	4.30	4.39	4.34	4.44
Days (Pupal to Eclosion)	Std. Error	0.05	0.05	0.05	0.05	0.04	0.05
Days (Pupal to Eclosion)	p value for Mann Whitney test to RF		**< 0.0001**	**< 0.0001**	**< 0.0001**	**< 0.0001**	**< 0.0001**

Supplemental Table S4B. Larval survival on CDF (n ≥ 65, 3 replicates, 20-25 larvae for each replicate)**.** One-tailed Fisher's exact tests were performed with the total counts, not the mean values. Statistically significant p values are labeled with bold text.

		RF	CDF^{100K}	CDF^{200K}	CDF^{300K}	CDF^{400K}	CDF^{500K}
Eclosed % (Hatch to Eclosion)	Mean	89.7	70.3	81.0	77.7	92.3	73.3
Eclosed % (Hatch to Eclosion)	Std. Error	5.2	7.3	6.7	3.8	1.5	6.7
Eclosed % (Hatch to Eclosion)	p value for Fisher's exact test to RF		**0.0025**	0.1118	0.0544	0.3816	**0.0202**
Survival % (Hatch to Pupal)	Mean	94.7	84.0	90.3	91.7	92.3	93.3
Survival % (Hatch to Pupal)	Std. Error	5.3	6.0	7.3	6.0	1.5	6.7
Survival % (Hatch to Pupal)	p value for Fisher's exact test to RF		**0.0485**	0.2652	0.3817	0.5000	0.3719
Survival % (Pupal to Eclosion)	Mean	95.0	83.3	89.7	85.2	100.0	78.3
Survival % (Pupal to Eclosion)	Std. Error	5.0	3.2	0.2	4.5	0.0	1.7
Survival % (Pupla to Eclosion)	p value for Fisher's exact test to RF		**0.0217**	0.2207	0.0592	0.1250	**0.0185**
