# Supplementary material for: Development and Characterization of a Chemically Defined Food for Drosophila
Source: PLoS One. 2013 Jul 2;8(7):e67308. doi: 10.1371/journal.pone.0067308 (PMC3699577; doi:10.1371/journal.pone.0067308)
Supplement: Table S5 — Effect of macro-nutrient deprivation on adult longevity and egg-lay. (PDF) [file pone.0067308.s007.pdf]

Supplemental Table S5A. Effect of macro-nutrient deprivation on adult longevity (n = 40, 4 replicates, 10 pairs of flies for each replicate)**.** Statistically significant p values are labeled with bold text.

		CDF^{400K}	$CDF^{400K\text{-}AA}$	$CDF^{400K\text{-}Carb}$	$CDF^{400K\text{-}Fat}$
Female median survival	Days	35.0	20.5	4.0	26.0
Female median survival	p value for Mantel-Cox test to CDF^{400K}		**< 0.0001**	**< 0.0001**	**0.0002**
Female median survival	p value for Mantel-Cox test to $CDF^{400K\text{-}AA}$			**< 0.0001**	**< 0.0001**
Female median survival	p value for Mantel-Cox test to $CDF^{400K\text{-}Carb}$				**< 0.0001**
Male median survival	Days	41.0	19.0	2.5	35.0
Male median survival	p value for Mantel-Cox test to CDF^{400K}		**< 0.0001**	**< 0.0001**	**0.0241**
Male median survival	p value for Mantel-Cox test to $CDF^{400K\text{-}AA}$			**< 0.0001**	**< 0.0001**
Male median survival	p value for Mantel-Cox test to $CDF^{400K\text{-}Carb}$				**< 0.0001**

Supplemental Table S5B. Effect of macro-nutrient deprivation on egg-lay (4 replicates, 10 pairs of flies for each replicate)**.** Statistically significant p values are labeled with bold text.

		CDF^{400K}	$CDF^{400K\text{-}AA}$	$CDF^{400K\text{-}Carb}$	$CDF^{400K\text{-}Fat}$
Total egg-lay (7 days)	Mean	81.8	18.7	28.3	80.7
Total egg-lay (7 days)	Std. Error	3.6	2.5	3.0	5.1
Total egg-lay (7 days)	p value for Mann Whitney to CDF^{400K}		**0.0286**	**0.0286**	0.7715
Total egg-lay (7 days)	p value for Mann Whitney to $CDF^{400K\text{-}AA}$			**0.0286**	**0.0286**
Total egg-lay (7 days)	p value for Mann Whitney to $CDF^{400K\text{-}Carb}$				**0.0286**
